# Under-Researched Demographics: Heavy Episodic Drinking and Alcohol-Related Problems Among Asian Americans

**DOI:** 10.35946/arcr.v38.1.03

**Published:** 2016

**Authors:** Derek Kenji Iwamoto, Aylin Kaya, Margaux Grivel, Lauren Clinton

**Affiliations:** Derek Kenji Iwamoto, Ph.D., is an assistant professor; Aylin Kaya is a doctoral student; Margaux Grivel is a project manager; and Lauren Clinton is a research assistant in the Department of Psychology, University of Maryland–College Park, College Park, Maryland

Historically, Asian Americans have reported lower rates of alcohol misuse compared with other racial/ethnic groups (Substance Abuse and Mental Health Services Administration 2009; Wechsler et al. 2000). However, epidemiological data illustrates that heavy episodic drinking and alcohol abuse are significant and increasing among U.S.-born Asian-American young adults ages 18–25 (Grant et al. 2004). Within one decade alone, the prevalence of alcohol abuse increased fivefold among Asian Americans, from 0.74 percent in 1991–1992 to 3.89 percent in 2001–2002 (Grant et al. 2004). Moreover, recent studies have identified high-risk subgroups of Asian-American young adults who engage in higher rates of heavy episodic drinking compared with their Asian-American peers (Iwamoto et al. 2010). Additionally, some U.S.-born Asian-American ethnic subgroups may engage in heavy episodic drinking at comparable rates to high-risk groups (e.g., college fraternity members) in the general population (Iwamoto et al. 2011*b*). Despite this growing concern, Asian Americans are perceived as a low-risk group with respect to alcohol problems, partially because of the “model minority” myth and the stereotype of Asian Americans generally being well assimilated to U.S. culture, being financially and academically successful, and with low levels of psychological distress (Gupta et al. 2011).

This general perception, which is largely upheld by the research community, hinders our understanding of the specific alcohol-related problems experienced by this population. However, given that Asian Americans are the fastest-growing racial group in the United States (Le 2010), it is crucial to understand the determinants and mechanisms of risk among Asian Americans. This article reviews the research over the last 15 years pertaining to Asian Americans’ alcohol use. Specifically, it highlights the role of genetic factors (e.g., alcohol dehydrogenase [ADH] and aldehyde dehydrogenase genes [ALDH]) as well as of sociocultural factors (e.g., physiological and cognitive expectancies, acculturation, enculturation, discrimination, mental health problems, and gender socialization) on heavy episodic drinking and alcohol-related problems in this demographic.

## Genetic Factors

Two genetic factors that have been significantly associated with alcohol use and related problems include specific variants (i.e., alleles) of the genes encoding certain ADH (ADH1B) and ALDH (ALDH2) enzymes. The *ADH1B* gene encodes an enzyme that metabolizes ethanol into acetaldehyde (Eng et al. 2007). One allele of this gene (i.e., ADH1B*2) encodes an enzyme that accelerates the oxidation of ethanol, resulting in a buildup of acetaldehyde (Borson and Li 1986; Eng et al. 2007; Eriksson 2001). High levels of acetaldehyde can create a heightened and unpleasant response to alcohol characterized by facial flushing, headache, and nausea (Wall et al. 2005), thereby making alcohol consumption unpleasant and thus protecting against high consumption and, consequently, risk of alcohol use disorder. Luczak and colleagues’ (2006) meta-analysis suggested that Asian individuals who are the most protected from alcohol abuse possess one or two copies of the ADH1B*2 allele. Specifically, Asians with two ADH1B*2 alleles were five times less likely to be dependent on alcohol than were those who did not possess this allele (Luczak et al. 2006).

The ADH1B*2 allele is found predominantly in certain subgroups of East Asians, including those of Japanese descent, of whom an estimated 81 percent carry at least one copy of this allele (Eng et al. 2007); Chinese descent (84 to 92 percent); and Korean descent (88 to 96 percent). The frequency of the ADH1B*2 allele in East Asians is comparable, albeit not precisely matched, to the rates of the ALDH2*2 allele, which encodes an inactive variant of ALDH2 (Eng et al. 2007). This suggests that although there is some overlap between those with the protective alleles of *ADH1B* and *ALDH2*, many carry only one but not the other. Carrying only one of the protective alleles still can reduce risk of alcohol use disorder. Thus, Asian Americans with a fully active *ALDH2* gene (ALDH2*1) who additionally possess the protective high-activity *ADH1B* allele (ADH1B*2) were 80 percent less likely to be alcohol dependent compared with Asian Americans who possessed the standard alleles of both enzymes (Luczak et al. 2006; Whitfield 2002). This finding indicates that ADH1B*2 may be protective against alcohol dependence even in the presence of the fully active *ALDH2* allele, suggesting even mild discomfort experienced because of alterations in acetaldehyde metabolism may prevent overindulgence in alcohol.

ALDH2 is responsible for mediating the oxidation of acetaldehyde generated by the actions of ADH into acetate (Bosron and Li 1986). Individuals carrying the reduced-activity *ALDH2* variant (ALDH2*2) metabolize acetaldehyde at a much slower rate or not at all, resulting in similar unpleasant symptoms after alcohol consumption to those associated with the ADH1B*2 allele (Crabb et al. 2004; Hendershot et al. 2009; Thomasson et al. 1993). The reduced-activity ALDH2*2 allele most commonly is found in people of East-Asian descent (i.e., Chinese, Japanese, and Korean), meaning these subgroups theoretically are most protected against alcohol abuse (Eng et al. 2007). Among subpopulations in the United States, the influence of ALDH2*2 is especially relevant for Asian Americans, because 30 to 50 percent of these individuals (Goedde et al. 1992) possess this enzyme deficiency that provokes physical irritation and discomfort (Hendershot et al. 2009; Thomasson et al. 1993). Given the high prevalence of the inactive *ALDH2* allele among Asian Americans, numerous studies have investigated its role in drinking behaviors in this population. These analyses have revealed that people carrying the ALDH2*2 allele are protected against alcohol abuse, especially those of Han Chinese and/or Japanese ethnicities (Luczak et al. 2006).

Although the ADH1B*2 and ALDH2*2 alleles both can serve as protective factors against alcohol abuse, they do not seem to eliminate alcohol consumption altogether. Wall and colleagues (2001) found that although college students with fully active *ALDH2* alleles were significantly more likely to be regular drinkers (78 percent) than those carrying an inactive allele, 58 percent of students with an inactive ALDH2*2 allele still were regular drinkers. Thus, even if possession of the inactive allele reduces the likelihood of alcohol consumption, it by no means provides full protection. In fact, the incidence of alcoholism in South Korea and Japan as well as of high-risk drinking among young adult Asian Americans is relatively high, even in people possessing an inactive ALDH2 enzyme (Higuchi et al. 1996; Kim et al. 2010; Wall et al. 2001; Yokoyama et al. 2003). Thus, beyond these genetic factors, sociocultural factors may influence the risk of alcohol use and related problems in this population (Doran et al. 2007; Luczak et al. 2004; Lum et al. 2009).

## Social–Cognitive Factors

### Alcohol Expectancies

One social–cognitive factor that seems to be associated with genetic factors, including the protective variants ALDH2*2 and ADH1B*2, is alcohol expectancies, or the cognitions and beliefs about the positive, negative, and physiological effects of alcohol use. Theoretically, alcohol sensitivity may influence alcohol-related learning processes, including development of alcohol expectancies, through differences in physiological responses to alcohol (Hendershot et al. 2009). Hendershot and colleagues (2009) developed a measure assessing physiological expectancies—that is, the anticipation that drinking excessively will result in negative physiological responses such as nausea, flushing, and dizziness—and subjective response to alcohol. The analyses revealed that individuals carrying the ALDH2*2 allele were more likely to have negative physiological alcohol expectancies and, thus, engage in lower alcohol use. Consequently, individuals who hold negative physiological alcohol expectancies and possess the inactive *ALDH2* allele are at substantially lower risk for alcohol abuse (Goldman et al. 2006; Hendershot et al. 2009). However, although negative physiological expectancies may be a prominent factor in risk of alcohol use, abuse, and dependence, especially in conjunction with genetic factors, other sociocultural factors further explicate Asian Americans’ drinking patterns.

### Acculturation

Acculturation is a multidimensional process that occurs when immigrants and people raised in immigrant households experience the merging of cultural norms, values, and behaviors from the heritage culture and receiving culture (Phinney 2003; Schwartz et al. 2010). Acculturation has been studied extensively in relation to alcohol use among Asian Americans. Thai and colleagues (2010) used data from the National Longitudinal Study of Adolescent Health to investigate the influence of acculturation, peer substance use, and academic achievement on alcohol use in Asian-American adolescents compared with other racial/ethnic groups. The study determined that acculturation, although not as robust a predictor of alcohol use as peer substance use and academic achievement, was more pertinent in Asian Americans than in other U.S. racial/ethnic groups. Specifically, people who were more acculturated were at greater risk for alcohol use. Similarly, Hahm and colleagues (2003) reported that Asian Americans who spoke English at home and were born in the United States (i.e., had a higher level of acculturation) were three times more likely to use alcohol compared with those who were less acculturated.

These findings highlight the possible stress that coincides with the acculturation process. Many people experience acculturative stress, defined as a significant reduction in physical, psychological, and social health related to the challenges of acculturation (Berry et al. 1987). Specific stressors include conflicting family values and expectations, having to learn a new language, experiencing discrimination, and struggling to adopt a new culture (Szapocznik et al. 1989). Because acculturative stress places people at increased risk for psychological distress, it also may encourage problematic coping behaviors, including heavy alcohol use or other self-medicating behaviors (Unger et al. 2009). The association between acculturative stress and heavy drinking has been identified among Asian-American samples. For example, one study (Park et al. 2014) investigating the impact of acculturation and related stress on alcohol use in Chinese, Filipino, and Vietnamese samples suggested that general acculturative stress was significantly related to alcohol use in Vietnamese-American participants.

One proxy of acculturation that has been studied in relationship to Asian-American drinking patterns is nativity, or whether an individual was born in the United States. Nativity has been identified as an important factor that helps explain within-group differences in drinking patterns among Asian Americans. According to the National Epidemiological Survey of Alcohol and Related Conditions (NESARC), U.S.-born Asian Americans were more likely to report alcohol abuse and alcohol dependence, as defined by the *Diagnostic and Statistical Manual of Mental Disorders, 4th Edition*, compared with foreign-born Asians (Breslau and Chang 2006). Similarly, a study of one of the largest samples of U.S.-born Asian-American young adults (*N* = 1,575) revealed that heavy episodic drinking and alcohol-related problems generally were higher in this group compared with studies that aggregate data for all Asian-American groups regardless of immigration and generational status (Iwamoto et al. 2012). The same study also investigated possible Asian-ethnic-group differences in drinking patterns and, consistent with other studies, found that individuals of Japanese, Filipino, South-Asian, multi-Asian (i.e., having parents from different Asian ethnic groups), and Korean descent reported higher rates of drinking compared with people of Chinese and Vietnamese descent (Duranceaux et al. 2008; Lum et al. 2009). Researchers have hypothesized that drinking rates may be higher among Japanese-and Filipino-American young adults because these groups tend to be more acculturated and, as a result, often have beliefs and values similar to their White-American counterparts (Chen et al. 1999). In contrast to this hypothesis, Hendershot and colleagues (2008) identified acculturation as a protective factor against alcohol abuse, specifically among Korean Americans. The researchers theorized that this trend was related to the cultural differences in alcohol use between Korea and other countries. Thus, Korea, in particular, has more permissive attitudes toward alcohol use and higher prevalence rates of alcohol use disorder compared with the United States. Taken together, research in this area has highlighted the significant within-group differences among Asian Americans and underscores the importance of analyzing U.S.- and foreign-born Asian Americans independently.

### Ethnic Drinking Cultures

In light of the well-documented heterogeneity in drinking patterns among Asian-American ethnic groups, Cook and colleagues (2009, 2012, 2013) have taken a novel and culturally focused approach to understanding problem drinking among this group. These investigators have conducted a number of key studies investigating culturally focused factors, including ethnic drinking cultures, or the “drinking cultures of the Asian countries of origin” (Cook et al. 2012, p. 340). The concept of ethnic drinking cultures illustrates how Asian Americans who are descendants of Asian countries with high per capita alcohol consumption (e.g., South Korea and Japan) may be at higher risk of problematic alcohol use compared with individuals from ethnic drinking cultures with low per capita alcohol consumption (e.g., Malaysia). Several key studies strongly support the influence of ethnic drinking cultures on drinking behaviors among Asian Americans and highlight how this factor may help explain unique variance in alcohol-use patterns above and beyond acculturation. Cook and colleagues (2009) revisited the role of acculturation in alcohol use patterns among Korean-American adolescents and found that after controlling for factors such as age, amount of spending money available, number of peers who drink, and social affiliation (i.e., Korean vs. non-Korean affiliations), no significant associations between acculturation and alcohol existed. In another study using Wave 2 of the NESARC, Cook and colleagues (2012) demonstrated that Asian Americans from ethnic drinking cultures with high per capita alcohol use were more likely to engage in heavier use and be current drinkers compared with individuals from drinking cultures with lower per capita use. Moreover, ethnic drinking cultures with high per capita alcohol use were associated with higher levels of intoxication, alcohol abuse, and alcohol dependence symptoms among foreign-born young-adult Asian Americans (Cook et al. 2013). These observations should encourage researchers to reevaluate the acculturation-centered approach in investigating alcohol use behavior in immigrant populations and to consider the complexity of Asian Americans’ experiences when researching other potential sociocultural factors in relation to alcohol use among this group.

### Enculturation

Another relevant cultural factor for Asian Americans is enculturation, or the adherence to a heritage culture’s traditional values, which is a separate and distinct process from acculturation. Thus, greater endorsement of the values of the heritage culture does not imply decreased adherence to the values of the receiving culture (Kim 2007; Miller et al. 2011). Enculturation is a central aspect of social identity and cultural adaptation among Asian Americans and arguably the primary process involved in identity development among this ethnic minority group (Weinreich 1999, 2009). Therefore, when studying drinking behaviors in Asian Americans, it is crucial to gain a firm understanding of the country of origin’s cultural values that influence alcohol use and abuse in this population. Kim and colleagues (2001) identified several distinct cultural values that are central to many Asian cultures, including collectivism, filial piety, humility, personal restraint, and emotional suppression. Filial piety, or respect for one’s parents, has been found to have a significant direct protective effect against alcohol initiation in Asian-American adolescents (Shih et al. 2012). Moreover, alcohol resistance self-efficacy (e.g., the ability to resist the pressure to use alcohol) and positive alcohol expectancies both mediated the effect of parental respect on alcohol initiation in this group. The investigators theorized that parental respect reduces alcohol initiation both by enabling individuals to develop the necessary skills to combat alcohol-use pressure and by reducing positive alcohol expectancies.

Although research by Shih and colleagues (2012) generated evidence linking distinct cultural values with alcohol-use behavior among Asian Americans, other studies have found no such relationships. Two studies examining broad Asian values (e.g., a global unidimensional measure called the Asian Values Scale that included collectivism, filial piety, and restricting emotions) and heavy episodic drinking among young adult Asian-American men (Liu and Iwamoto 2007) and Asian-American women (Iwamoto et al. 2011*a*) found no significant effect of Asian cultural values on drinking behaviors. This lack of a significant relationship could be a consequence of the assessment approach of the Asian Values Scale (Kim et al. 1999) that was used in these two studies. Thus, this instrument measures cultural values unidimensionally—that is, although the scale assesses six separate aspects of cultural values (i.e., conformity to norms, family recognition through achievement, emotional self-control, collectivism, humility, and filial piety), the six scores are summed together to create a global aggregated score. Such a global score may be too generalized to detect the protective/ risk effects of the individual cultural values (e.g., filial piety). Future studies need to provide greater specificity to clarify the relationship between cultural values and drinking behaviors among Asian-American young adult samples.

Collectively, the literature exploring the roles of acculturation, nativity, and enculturation on heavy episodic drinking and alcohol use among Asian Americans has yielded inconsistent results. With the rates of alcohol use and abuse among Asian Americans rapidly rising (Grant et al. 2004; Iwamoto et al. 2010), it is imperative to conduct further research to clarify the roles of additional sociocultural variables, including discrimination and mental health, on alcohol use behaviors in this group.

### Discrimination

Perceived discrimination has been found to be a cause for self-medication (Khantzian 1985), implying a plausible link between experienced negative and unfair treatment of Asian Americans and alcohol use. Asian Americans seem to experience a significant amount of discrimination at comparable rates with other racial groups (Iwamoto and Liu 2010; Yoo et al. 2010). For example, Asian Americans may be treated or perceived as perpetual foreigners even though many of them were born in the United States, or may receive differential treatment because of their racial/ethnic characteristics (Yoo et al. 2010). Analyses among Asian-American adults revealed that a significant association exists between racial discrimination and alcohol use, even after controlling for gender, nativity, and language use (Yoo et al. 2010). Additionally, Chae and colleagues (2008) found that unfair treatment in the form of discrimination was associated with higher odds of having alcohol use disorder in both U.S.- and foreign-born Asian Americans. Perceived ethnic identification served as a possible buffer against such disorders, because higher levels of ethnic identification moderated the effects of discrimination on alcohol use disorder (Chae et al. 2008). Similarly, Gee and colleagues (2007) examined the relationship between perceived discrimination and alcohol dependence among a nationally representative sample of Filipino Americans. These investigators detected a twofold increased probability of alcohol dependence for every one-unit increase in reported unfair treatment (i.e., being treated differently because of their ethnicity, speaking a different language, or having an accent) (Gee et al. 2007). These findings suggest that Asian Americans may abuse alcohol to cope with the deleterious stressors of discrimination.

### Mental Health

In the general population, alcohol-related problems have been linked to mental health problems such as unspecified psychological distress and depression. A similar association seems to exist for Asian Americans (Grant et al. 2004; Windle and Davies 1999). Mental health problems are especially relevant for Asian-American young adults who have one of the highest rates of depressive symptomology (Iwamoto et al. 2011*a*; Kearney et al. 2005). Several studies examining Asian-American adolescents have revealed that depressive symptomology and suicidal ideation were significantly associated with problematic alcohol use (Nishimura et al. 2005; Otsuki 2003). Among young-adult Asian-Americans samples, depressive symptoms were associated with heavy episodic drinking and alcohol-related problems (Iwamoto et al. 2011*a*; Kim et al. 2014). Finally, depressive symptoms and mental health problems, including anxiety disorders and suicidal ideation, were related to heavy drinking among a nationally representative community sample of Asian-American women (Cheng et al. 2012). These associations are consistent with previous research indicating that poor mental health and alcohol abuse often co-occur (Canino et al. 2008). In particular, the association with mental health problems seems to be an especially relevant factor in explaining alcohol-use patterns for Asian-American women.

### Gender-Relevant Factors

Alcohol consumption is a gendered activity—that is, gender differences exist in drinking behavior, and cultural norms predispose men to generally drink more than women (Grant et al. 2004). However, alcohol use and alcohol-related problems seem to be a growing concern among Asian-American women. One study using a nationally representative sample of Asian-American adolescents revealed that in this population, girls engage in heavy episodic drinking more frequently than do boys (33.6 percent for girls vs. 30.6 percent for boys) (Hahm et al. 2004). It is possible that gendered and cultural expectations may exacerbate stress and encourage heavy drinking as a self-medicating behavior, given the unique gender expectations placed on Asian-American women (Pyke and Johnson 2003). These women often are perceived as hyperfeminine, passive, subservient, dutiful, and sexually exotic (Pyke and Johnson 2003). Accordingly, Asian-American women’s gendered experiences may present unique risks for alcohol use and abuse, and research should focus on gender-relevant factors to help explain Asian-American women’s alcohol use.

One theoretically promising gender-relevant factor includes conformity to feminine norms, or endorsement of beliefs, expectations, and values of what it means to be a woman (Mahalik et al. 2005). Feminine norms may help explain sex differences and within-group variability in alcohol use and related problems among women. Adherence to feminine norms may be particularly salient for Asian-American women who have to contend with the demands of acculturative factors as well as of the culture of origin, which generally emphasizes conforming to traditional notions of femininity. Because of the expectation of hyperfemininity, Asian-American women may strongly internalize feminine norms, potentially resulting in high pressure to meet gender-relevant standards set out of reach by society (Levant 1996). This stress of attempting to meet unattainable, heavily emphasized notions of femininity may result in negative health symptomology (Boskind-Lodahl 1976) and ultimately may influence heavy drinking as a means of self-medication and coping with gender strain.

Similarly to Asian-American women, Asian-American men experience racialized and gender-based stereotypes, including being perceived as awkward, sexually inadequate, and perpetual foreigners (Wong et al. 2012). For Asian-American men, one way of reinforcing masculinity may be through alcohol use, likely because drinking large quantities of alcohol has been linked to traditional notions of masculinity, including power and control (Iwamoto 2010). Masculine norms, like feminine norms, are multidimensional and describe the socially constructed beliefs, values, and expectations of what it means to be a man in contemporary U.S. society (Mahalik et al. 2003). These norms include (1) striving to win at all costs; (2) being a playboy, or demonstrating sexual prowess; (3) showing emotional control; (4) engaging in risk taking; (5) exhibiting an inclination towards violence and physical aggression; (6) asserting dominance; (7) being self-reliant; (8) prioritizing work; (9) having power over women; (10) presenting oneself as heterosexual; and (11) pursuing importance or high status. Men are expected to ascribe to these norms in order to prove and display their manliness.

It is important to understand these gender-relevant factors in relation to men’s alcohol use, because drinking is viewed as a symbol of manliness in the United States. As a result, men who strive to endorse specific masculine norms may engage in more problematic drinking patterns (Lemle and Mishkind 1989). Research has supported this notion; in particular, several studies suggest that power over women (Liu and Iwamoto 2007) and being a playboy (i.e., desiring to have multiple sexual partners), risk-taking, and striving to win (Iwamoto et al. 2011*b*, 2014) heighten the risk of problematic drinking and alcohol-related problems. Consistent with masculine-norms theory (Courtenay 2000; Levant 1996) men may attempt to prove their masculinity by consuming large quantities of alcohol, drinking as many alcoholic beverages as fast as they can, and demonstrating how much alcohol they can “hold” or tolerate (Iwamoto 2010). In particular, men who want to have multiple sex partners or adhere to the playboy norm, as well as those who endorse self-reliance norms, are at increased risk for reporting alcohol-related consequences (Iwamoto et al. 2011*b*). However, other masculine norms (e.g., primacy of work) may protect against drinking to intoxication, because men who orient themselves toward this norm prioritize their work and thus may not want to drink heavily.

Thus, multidimensional gender norms seem to serve as both risk and protective factors for heavy episodic drinking and subsequent alcohol-related problems (Iwamoto et al. 2011*b*; Lemle and Mishkind 1989). Although gender-relevant research among Asian Americans to date has yielded promising results, future research must continue to examine the intersection of gender and culture in relation to problem drinking.

## Summary

Asian Americans represent the fastest-growing population in the United States (Le 2010). At the same time, there is evidence that problematic drinking rates are increasing among young-adult Asian Americans (Grant et al. 2004). Accordingly, it is essential to understand the etiological determinants and mechanisms of risk that may help explain this growth in problematic alcohol use among this group. The high prevalence of the ALDH2*2 and ADH1B*2 alleles in a large percentage of Asian subgroups has been studied as a potential protective factors against alcohol abuse, yet some individuals who possess these genes still engage in problematic alcohol use (Wall et al. 2001). Other social and psychological factors may account for this discrepancy. Thus, some factors, such as negative physiological alcohol expectancies, are protective against alcohol abuse in this population (Hendershot et al. 2009). Sociocultural factors such as acculturation and nativity also may help explain drinking patterns among this group.

The literature suggests that vast and significant within-group differences exist among Asian Americans, such that individuals who were born in the United States and/or are more acculturated are at elevated risk for alcohol abuse and related problems (Hahm et al. 2003). Differences also have been observed among Asian-American ethnic subgroups, with some groups (e.g., Japanese, Korean, and multi-Asian Americans) reporting higher rates of drinking compared with others (e.g., Chinese and Vietnamese Americans) (Iwamoto et al. 2012). Furthermore, Asian Americans who report higher levels of depressive symptoms, psychological distress, and perceived discrimination seem to be at a heightened risk for abusing alcohol (Iwamoto et al. 2011*a*; Nishimura et al. 2005; Yoo et al. 2010). Finally, an emerging body of research examining gender-relevant factors, including feminine and masculine norms, may help explain within-group differences among Asian-American women and men. Thus, traditional norms that may directly pertain to hyperfemininzed Asian-American women, including modesty and sexual fidelity, may protect against heavy episodic drinking (Young et al. 2005). Conversely, the risk for heavy episodic drinking may be enhanced in men who strive to demonstrate traditional notions of masculinity through risk-taking and endorsement of playboy norms (Iwamoto et al. 2010).

Although this review has illustrated the contemporary state of research on alcohol use among Asian Americans, it also highlights the significant limitations in this literature. Many of the studies reviewed here have used cross-sectional data, which do not allow researchers to infer causality between the various sociocultural factors and problematic alcohol use. One way of addressing this gap in the existing literature may be to implement longitudinal designs to further understand how the temporal relationship between sociocultural factors, including acculturation and gender norms, may impact alcohol use and alcohol-related problem trajectories. There also is a pressing need to develop greater understanding of within-group differences among U.S.-born and foreign-born Asian Americans as well as among as specific ethnic groups. To date, epidemiological research has largely neglected to examine these significant discrepancies. Given the growing prevalence of alcohol use and alcohol-related problems among Asian-American women (Grant et al. 2004; Iwamoto et al. 2010), studies also should focus on this group and explore how the intersection of gender and culture may influence alcohol use. Finally, the majority of research on this population has been conducted in college samples; therefore, it is important to also examine community samples, including U.S.-born young adults who are not attending college and older adult Asian-American populations.
